# Enzyme-linked immunosorbent assay for the quantitative/qualitative analysis of plant secondary metabolites

**DOI:** 10.1007/s11418-017-1144-z

**Published:** 2017-11-21

**Authors:** Seiichi Sakamoto, Waraporn Putalun, Sornkanok Vimolmangkang, Waranyoo Phoolcharoen, Yukihiro Shoyama, Hiroyuki Tanaka, Satoshi Morimoto

**Affiliations:** 1 Department of Pharmacognosy, Graduate School of Pharmaceutical Sciences, Kyushu University, 3-1-1 Maidashi, Higashi-ku, Fukuoka, 812-8582 Japan; 2 Research Group for Pharmaceutical Activities of Natural Products using Pharmaceutical Biotechnology (PANPB), Faculty of Pharmaceutical Sciences, Khon Kaen University, Khon Kaen, 40002 Thailand; 3 Department of Pharmacognosy and Pharmaceutical Botany, Faculty of Pharmaceutical Sciences, Chulalongkorn University, 254 Phayathai Rd. Pathumwan, Bangkok, 10330 Thailand; 4 Department of Pharmacognosy, Faculty of Pharmaceutical Sciences, Nagasaki International University, 2825-7 Huis Ten Bosch, Sasebo, Nagasaki, 859-3298 Japan

**Keywords:** Antibodies, Enzyme-linked immunosorbent assay (ELISA), Hapten, Plant secondary metabolites

## Abstract

Immunoassays are antibody-based analytical methods for quantitative/qualitative analysis. Since the principle of immunoassays is based on specific antigen–antibody reaction, the assays have been utilized worldwide for diagnosis, pharmacokinetic studies by drug monitoring, and the quality control of commercially available products. Berson and Yalow were the first to develop an immunoassay, known as radioimmunoassay (RIA), for detecting endogenous plasma insulin [1], a development for which Yalow was awarded the Nobel Prize in Physiology or Medicine in 1977. Even today, after half a century, immunoassays are widely utilized with some modifications from the originally proposed system, e.g., radioisotopes have been replaced with enzymes because of safety concerns regarding the use of radioactivity, which is referred to as enzyme immunoassay/enzyme-linked immunosorbent assay (ELISA). In addition, progress has been made in ELISA with the recent advances in recombinant DNA technology, leading to increase in the range of antibodies, probes, and even systems. This review article describes ELISA and its applications for the detection of plant secondary metabolites.

## Introduction

Since the development of radioimmunoassay (RIA) in 1960, there has been a rapid increase in immunoassay techniques using radioactive labels [1]. However, radioactive labels have been gradually replaced with enzyme labels because of safety concerns associated with radioactivity since the study by Avrameas in 1969, who coupled antigens or antibodies and enzymes using glutaraldehyde [2]. Currently, ELISA has a higher number of immunoassays compared to RIA.

Plant secondary metabolites are plant-produced organic compounds that play an important role in the defense of plants against herbivores, pests, and pathogens, as well as in their adaptation to the environment, although they are not directly involved in the growth and development of organisms [3, 4]. Because of their diverse functions, there has been a dramatic increase in their demand in pharmaceuticals, cosmetics, and pesticides, as well as in food additives [5]. Quality control of these commercial products containing secondary metabolites is crucial as the quality directly affects their potential activity. In addition, Cragg and Newman recently reported that 34% of the currently used drugs originate from natural products [6]. Meanwhile, simple, selective, and sensitive analytical techniques are also required in pharmacodynamic studies for monitoring effective concentration, side effects, and metabolism, leading to a better quality of life for patients. Thus far, various analytical methods have been developed for such purposes, mainly based on high-performance liquid chromatography (HPLC). However, ELISA exhibits several advantages over such techniques because of its simplicity, selectivity, and sensitivity.

The basic facts about ELISA and its practical use for measuring plant secondary metabolites are described in this review.

## General principle of ELISA

ELISA is based on the concept of antigen–antibody reactions, representing the chemical interaction between antibodies produced by the B cells of leukocytes and antigens. This specific immune response plays an important role in protecting the body from invaders such as pathogens and toxins. Hence, by exploiting this reaction, ELISA permits the highly sensitive and selective quantitative/qualitative analysis of antigens, including proteins, peptides, nucleic acids, hormones, herbicides, and plant secondary metabolites. To detect these molecules, an antigen or antibody is labeled using enzymes, the so-called enzyme immunoassay, in which alkaline phosphatase (ALP) [7], horseradish peroxidase (HRP) [8], and β-galactosidase [9–11] are commonly used. The antigen in the fluid phase is immobilized on a solid phase, such as a microtiter plate constituting rigid polystyrene, polyvinyl chloride, and polypropylene. Subsequently, the antigen is allowed to react with a specific antibody, which is detected by an enzyme-labeled secondary antibody. The development of color using a chromogenic substrate corresponds to the presence of the antigen. For instance, ALP hydrolyzes *p*-nitrophenyl phosphate to produce *p*-nitrophenol, which can be detected at 405 nm (yellow color), and HRP catalyzes the conversion of chromogenic substrates, e.g., 2,2′-azino-bis(3-ethylbenzothiazoline-6-sulfonic acid) diammonium salt, 3,3′,5,5′-tetramethylbenzidine, and *o*-phenylenediamine into colored products. By using chemiluminescent substrates such as chloro-5-substituted adamantyl-1,2-dioxetane phosphate and luminol for ALP and HRP, respectively, and fluorogenic substrates such as 4-methylumbelliferyl galactoside and nitrophenyl galactoside for β-galactosidase, even more sensitive detection can be achieved. These enzyme–substrate reactions are typically completed within 30–60 min, and the reaction stops with the addition of an appropriate solution, e.g., sodium hydroxide, hydrochloric acid, sulfuric acid, sodium carbonate, and sodium azide, for individual reactions [12, 13]. Finally, colored or fluorescent products are detected using a microtiter plate reader.

## Advantages and disadvantages of ELISA

Advantages and disadvantages of ELISA are summarized in Table 1. ELISA exhibits the following advantages: (i) Simple procedure. (ii) High specificity and sensitivity, because of an antigen–antibody reaction. (iii) High efficiency, as simultaneous analyses can be performed without complicated sample pre-treatment. (iv) Generally safe and eco-friendly, because radioactive substances and large amounts of organic solvents are not required. (v) Cost-effective assay, as low-cost reagents are used. However, ELISA exhibits the following disadvantages: (i) Labor-intensive and expensive to prepare antibody because it is a sophisticated technique, and expensive culture cell media are required to obtain a specific antibody. (ii) High possibility of false positive or negative results because of insufficient blocking of the surface of microtiter plate immobilized with antigen. (iii) Antibody instability because an antibody is a protein that requires refrigerated transport and storage.

**Table 1:** Advantages and disadvantages of ELISA

Advantages	Disadvantages
Simple procedure	Labor-intensive and expensive to prepare antibody
Easy to perform with simple procedure	Sophisticated techniques and expensive culture media are required
High specificity and sensitivity	High possibility of false positive/negative
ELISA is based on antigen–antibody reaction	Insufficient blocking of immobilized antigen results in false results
High efficiency	Antibody instability
Simultaneous analysis can be performed without complicated sample pre-treatment	Refrigerated transport and storage are required as an antibody is a protein
Generally safe and eco-friendly	
Radioactive substances and large amounts of organic solvent are not required	
Cost-effective assay	
Reagents are relatively low cost	

## Types of ELISA

### Direct ELISA

**Fig. 1:**
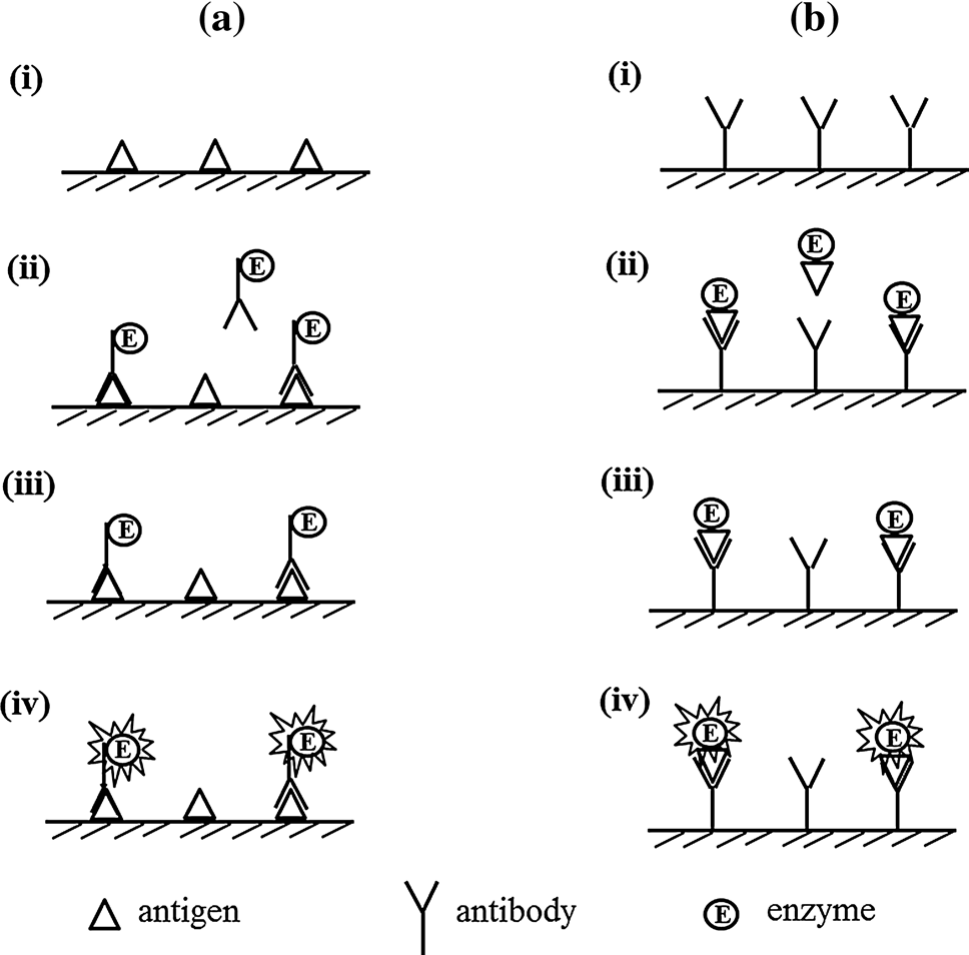
Direct ELISA to detect antigen (**a**) and antibody (**b**). (i) Attach antigen/antibody to solid phase. (ii) Incubate with enzyme-labeled antibody/antigen. (iii) Wash unbound enzyme-labeled antibody/antigen out. (iv) Develop color with substrate

In 1971, Engvall and Perlmann [14] and Van Weemen and Schuurs [15] were the first to develop direct ELISA (Fig. 1), which was the base style for other types of ELISA. Primarily, an antigen or an antibody is immobilized on the surface of microtiter plate. After the surface is blocked with other proteins (e.g., albumin, gelatin, casein, and skimmed-milk [13]) to avoid the non-specific adsorption of other proteins, the corresponding enzyme-labeled antibody or antigen is allowed to react with the immobilized targets, followed by color development with appropriate substrates. With an increasing amount of targets, the signal increases. Direct ELISA is suitable for the qualitative analysis of macromolecules.

### Competitive ELISA

**Fig. 2:**
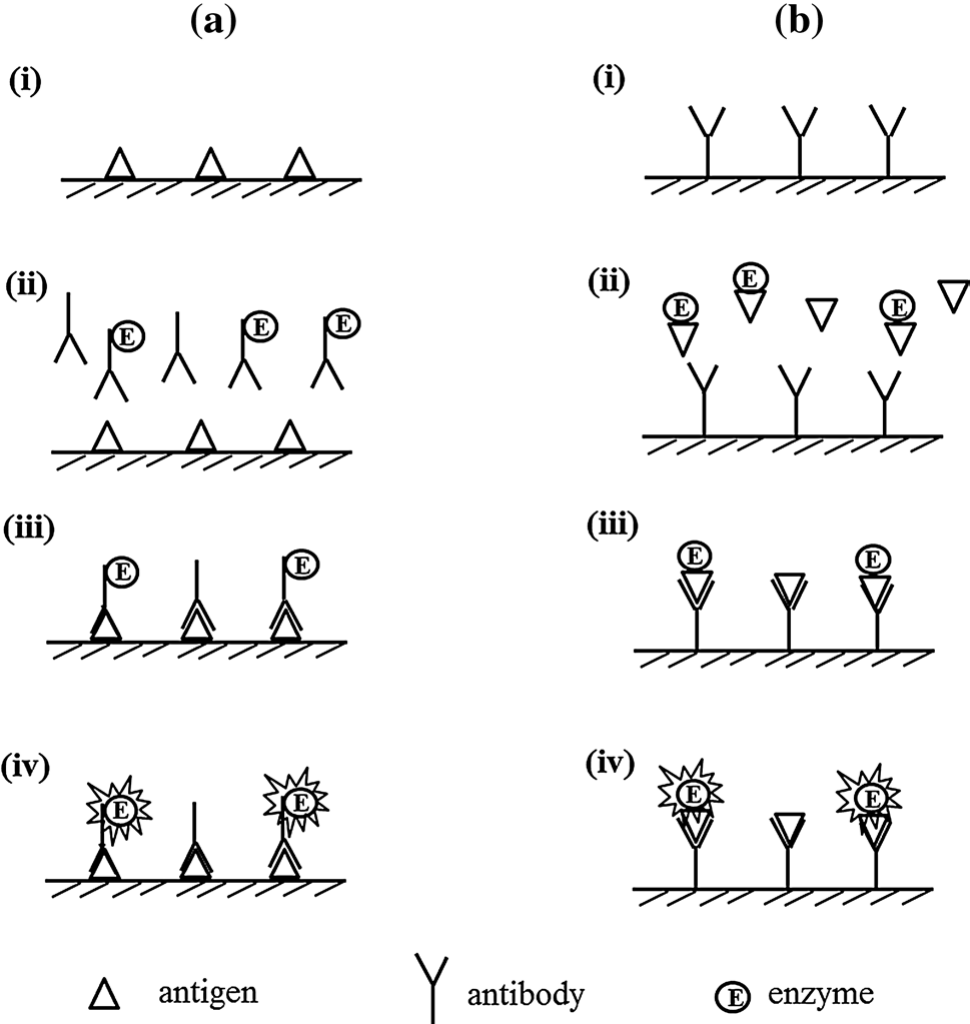
Competitive ELISA to detect antigen (**a**) and antibody (**b**). (i) Attach antigen/antibody to solid phase. (ii) Incubate antibody/antigen with enzyme-labeled antibody/antigen. (iii) Wash unbound enzyme-labeled antibody/antigen out. (iv) Develop color with substrate

In 1973, Belanger developed competitive ELISA (Fig. 2) to detect rat α-fetoprotein, which involved the development of indirect ELISA and sandwich ELISA [16]. The key event of competitive ELISA is the competitive reaction between targets (antigen or antibody) in the sample and enzyme-labeled targets (antigen or antibody) against corresponding immobilized antibody or antigen. To detect the antigen in competitive ELISA, an enzyme-labeled antigen is used to compete with the target antigens against the immobilized antibody (Fig. 2b). Hence, the higher the amount of antigen in the sample, the lower the amount of enzyme-labeled antigen that binds to the antibody. That is, with an increasing amount of target antigen, the signal decreases. In this case, competitive ELISA is suitable for measuring macromolecules only because a labeling enzyme is required to measure the antigen. If the antigen is a low molecular weight compound (e.g., hapten), resultant hapten–enzyme conjugates are not recognized by the immobilized antibody, leading to failure of the analysis. To detect the antibody, the antigen is immobilized, and the competition between the antibody in the sample and enzyme-labeled antibody is observed (Fig. 2a). In this case, both macromolecules and hapten can be detected when hapten is exposed on the surface of the microtiter plate.

Furthermore, detectable targets (antigen or antibody) can be changed depending on the competitors. When free antigen is used as competitor instead of unlabeled antibody in Fig. 2a, competitive reaction between free antigen and immobilized antigen against enzyme-labeled antibody can be observed, enabling the detection of free antigen (macromolecules and hapten) in the sample in this competitive system, and vice versa when free antibody is used instead of unlabeled antigen in Fig. 2b.

Direct and competitive ELISA methods are simple because only one antibody is required. However, the labeling step is required for each of the ELISA methods, possibly leading to inactivation of the antibody (Table 2).

**Table 2:** Characteristics of various types of ELISA

	Direct ELISA	Competitive ELISA	Indirect ELISA	Indirect competitive ELISA	Sandwich ELISA
Advantage	Simple because only one antibody is used		Higher sensitivity and versatility than direct methods owing to usage of PAb that recognizes different epitopes of primary antibody		High specificity as two antibodies possessing different epitopes are used
Disadvantage	Labeling antibody is necessary for each ELISA, which may result in inactivation of antibody		Nonspecific signal is induced through cross-reactivity of secondary antibody		To prepare two different antibodies is labor-intensive and expensive
Target	Macromolecules	Macromolecules (Hapten)	Macromolecules	Macromolecules (Hapten)	Generally macromolecules
Signal (as target antigen increase)	Increase	Decrease	Increase	Decrease	Increase

### Indirect ELISA

**Fig. 3:**
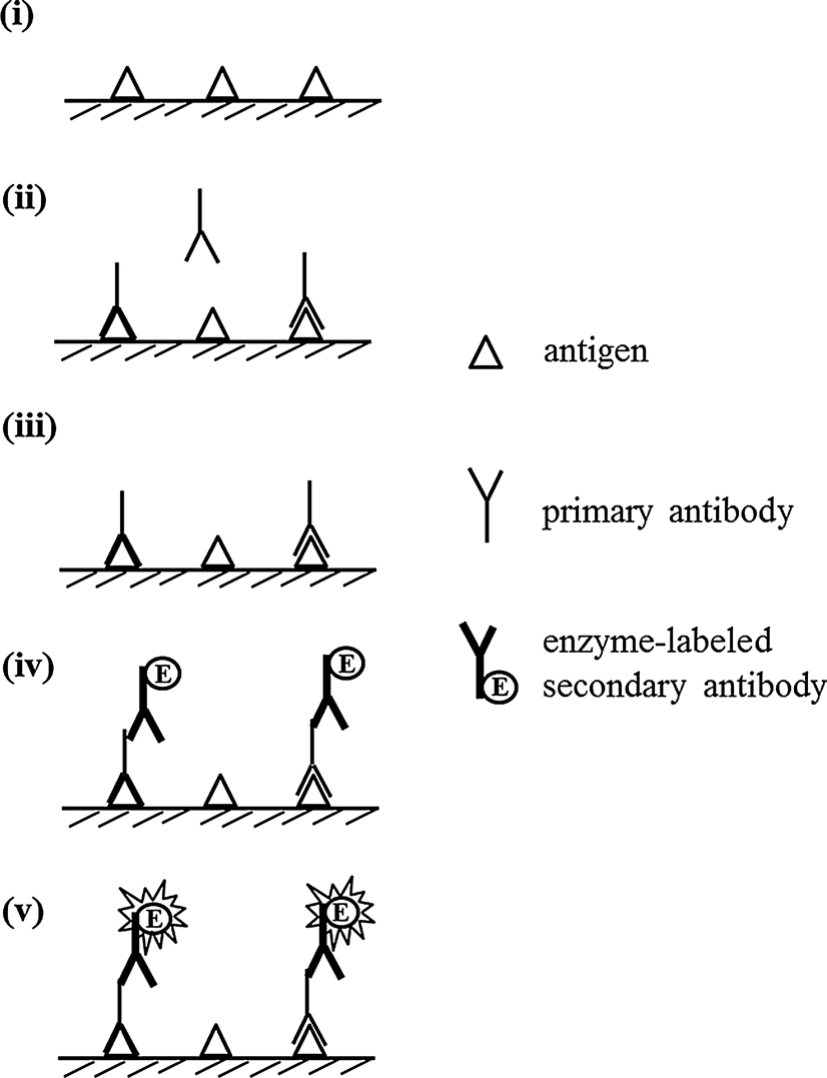
Indirect ELISA to analyze antibody. (i) Attach antigen to solid phase. (ii) Incubate with primary antibody. (iii) Wash unbound primary antibody out. (iv) Incubate with enzyme-labeled secondary antibody. (v) Develop color with substrate

Indirect ELISA systems have been developed on the basis of direct ELISA to evaluate the presence of antibody in antisera (Fig. 3) [17, 18]. The key step of this system is the two-binding process of the primary antibody and enzyme-labeled secondary antibody. That is, the target antigen is indirectly detected by the secondary antibody, which is labeled with the enzyme, or the so-called indirect ELISA. The antigen is primarily immobilized on the surface of the microtiter plate, which blocks the surface with blocking proteins as mentioned above. The primary antibody (in antisera) binding to the immobilized antigen is then allowed to react with the enzyme-labeled secondary antibody, followed by the development of color. The signal increases with an increasing amount of the immobilized target antigen. Indirect ELISA is suitable for measuring macromolecules. With the use of antisera as the primary antibody, the presence of a disease-associated antibody in the antisera can be evaluated; thus, indirect ELISA is effectively used to diagnose endocrine diseases [19, 20].

### Indirect competitive ELISA

**Fig. 4:**
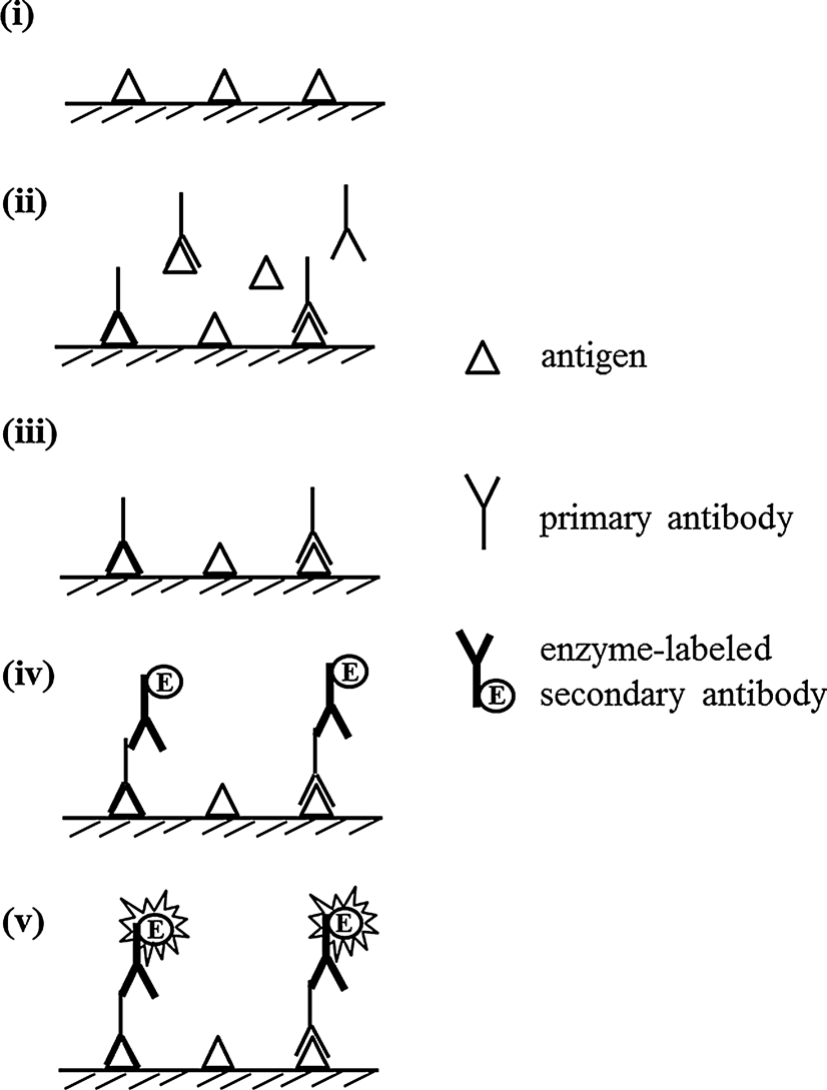
Indirect competitive ELISA to detect antigen. (i) Attach antigen to solid phase. (ii) Incubate free target antigen with primary antibody. (iii) Wash unbound free target antigen and primary antibody out. (iv) Incubate with enzyme-labeled secondary antibody. (v) Develop color with substrate

Indirect competitive ELISA (icELISA) involves the combination of indirect ELISA and competitive ELISA (Fig. 4). The target antigen is immobilized on a solid phase of the microtiter plate and is blocked. Subsequently, free target antigen and antibody are allowed to incubate and there is a competition between the immobilized antigen and free antigen against antibodies. The primary antibody that binds to the immobilized antigen is detected by the enzyme-labeled secondary antibody. Similar to the case in competitive ELISA, in icELISA, the signal decreases with increasing amount of the free antigen. icELISA can be applied for measuring both the macromolecules and hapten when hapten is exposed on the surface of the microtiter plate.

The use of enzyme-labeled secondary antibodies in indirect methods (e.g., indirect ELISA and icELISA) exhibit advantages over direct methods (direct and competitive ELISA) with respect to sensitivity and versatility [16]. Polyclonal antibody is a type of enzyme-labeled secondary antibody that recognizes different epitopes of the primary antibody, leading to increased sensitivity as compared to direct methods. In addition, a universal secondary antibody can be used if the original animal species of the primary antibody are unified. Thus, the secondary antibody is commercially available, leading to high versatility. Indirect ELISA clearly exhibits disadvantages with respect to the secondary antibody, i.e., the cross-reaction of the secondary antibody should be considered (Table 2).

### Sandwich ELISA

**Fig. 5:**
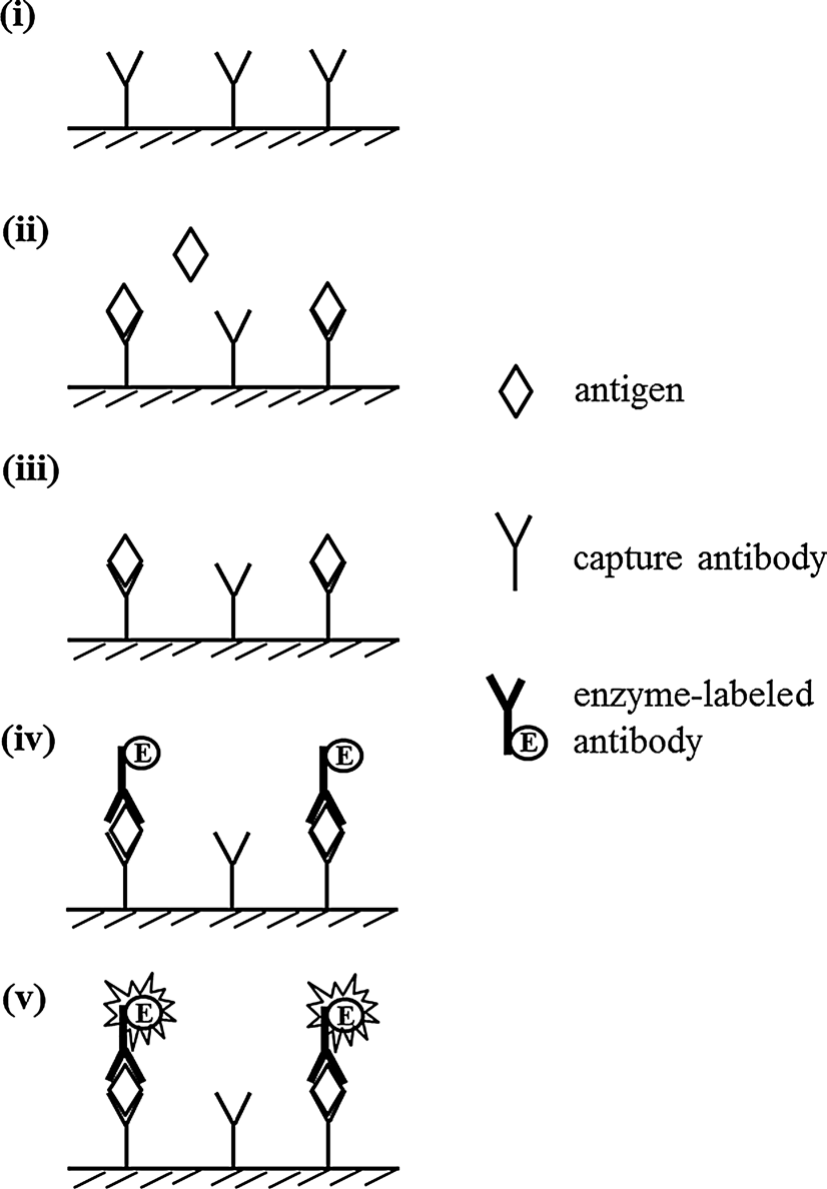
Sandwich ELISA for specific detection of antigen. (i) Attach capture antibody to solid phase. (ii) Incubate with target antigen. (iii) Wash unbound target out. (iv) Incubate with enzyme-labeled antibody. (v) Develop color with substrate

In this system, the target antigen is detected via anchoring between two antibodies, which recognize different epitopes, or the so-called sandwich system (Fig. 5) [16]. Sandwich ELISA starts from the immobilization of an antibody, called a capture antibody, on the microtiter plate. After blocking the plate surface to avoid non-specific adsorption of other proteins, the antigen in the sample is allowed to react with the immobilized capture antibody, and the antigen bound to the capture antibody is then sandwiched with an enzyme-labeled antibody for color development. This direct system can be modified to the indirect system by using primary and enzyme-labeled secondary antibodies. The signal increases with increasing amount of antigen. As two antibodies containing different epitopes are required against the target antigen, sandwich ELISA is generally suitable for measuring macromolecules with some exceptions. Ciguatoxins, which are produced in the marine dinoflagellate *Gambierdiscus toxicus*, are the major causative toxins of ciguatera seafood poisoning. Ciguatoxins are structurally classified as ladder-like polyethers with a molecular weight of 1111 Da. Oguri et al. divided these polyethers into two parts and prepared different monoclonal antibodies (MAbs) to individually recognize each part for constructing a sandwich ELISA system to measure ciguatoxins [21]. More recently, Boscolo et al. reported a sandwich ELISA method for marine biotoxins, e.g., palytoxins with a molecular weight of 2680 Da [22]. The sandwich was formed by using two antibodies obtained from the same antigen with different antibodies, i.e., MAb and PAb, which were used as the capture and primary antibodies, respectively.

A highly specific assay can be obtained via a sandwich system because of the use of two antibodies. However, it is an expensive and labor-intensive process to prepare two antibodies. In addition, one more step is required in the sandwich system because immobilization is necessary for capture antibody, which increases the assay time (Table 2).

### Open sandwich ELISA (OS-ELISA)

Advances in DNA technology have enabled the development of unique and interesting immunoassays based on the interaction of variable regions of heavy (V_H_) and light (V_L_) chains, which are binding regions for antigens [23]. In the presence of an antigen, the interaction between V_H_ and V_L_ regions is enhanced to form a ternary complex. In the aforementioned report, OS-ELISA started from coating of a solid-phase microtiter plate with streptavidin. After blocking, the V_L_ region conjugated with biotin was allowed to react with streptavidin to immobilize the V_L_ region. In the next process, the phage-displayed V_H_ region was incubated with hen egg lysozyme, which was used as an antigen. Finally, the phage-displayed V_H_ regions forming a ternary complex were detected by the HRP-labeled antibody to develop color. The obtained signal increases with increasing amount of antigen. Currently, this OS-ELISA has been modified to be more easy and effective, and several studies on OS-ELISA for measuring both the macromolecule and hapten have been reported [24–27].

## Types of antibody

In ELISA, any antibody can be used. In the first report of immunoassay developed by Berson and Yalow, PAb present in the antisera of immunized guinea pigs was used to detect human insulin [1]. However, issues related to the specificity of different batches were particularly concerning until the development of the MAb technology by Köhler and Milstein in 1975 [28]. In 1984, together with Niels Kaj Jerne, they were awarded the Nobel Prize in Physiology or Medicine. The emergence of MAb has helped in overcoming the issues with PAb. Since then, advances in DNA technology have enabled the production of recombinant antibodies, which include single-chain variable fragment (scFv) antibody, bispecific Bis-scFv, fragment antigen-binding (Fab) antibody, bispecific Fab_2_, trispecific Fab_3_, bivalent minibody, and multibody (diabody, triabody, and tetrabody) [29]. All of the aforementioned recombinant antibodies can be applied to ELISA, although the corresponding secondary antibodies need to be prepared.

## ELISA for plant secondary metabolites

### Specificity of antibody against hapten

Most of the useful plant secondary metabolites are low molecular weight compounds (i.e., hapten) with immense structural diversity, which are generally classified on the basis of their biosynthesis pathway [30, 31]. Hence, ELISA used for their analysis is the competitive type (competitive ELISA or icELISA) using MAb or PAb. When MAb is compared with PAb against hapten, MAb tends to exhibit higher specificity because PAb recognizes several epitopes, while MAb recognizes only one epitope. In addition, hybridoma cells secreting MAb exhibiting desirable characteristics can be screened. Pongkitwitoon et al. have prepared PAb against bioactive isoflavonoids, daidzin (DZ), by immunizing rabbits with DZ–bovine serum albumin (BSA) conjugates to develop icELISA [32]. By comparing the cross-reactivity (CR), which is the factor of specificity calculated by the ratio of IC_50_ for DZ to that for the test compounds, of PAb with that of MAb obtained from the same DZ–BSA conjugates [33], the specificity of MAb to DZ was greater than that of PAb (Table 3).

**Table 3:** Chemical structures of representative isoflavonoids and cross-reactivities (CRs) of PAb [32], MAb [33] produced from DZ–BSA conjugates prepared by NaIO_4_ oxidation method, and MAb [39] produced from DZ–cBSA conjugates obtained by Mannich reaction

Isoflavonoids	R_1_	R_2_	R_3_
Daidzin (DZ)	H	Glc–	H
Daidzein	H	H	H
Genistin	H	Glc–	OH
Genistein	H	H	OH
Puerarin	Glc–	H	H

Compound	CRs of PAb [32] (NaIO_4_ oxidation)	CRs of MAb [33] (NaIO_4_ oxidation)	CRs of MAb [39] (Mannich reaction)
Diadzin (DZ)	100	100	100
Daidzein	93.4	16.2	1.6
Genistin	49.0	82.4	0.044
Genistein	45.1	24.4	< 0.015
Puerarin	0.1	3.4	< 0.015

Apart from the types of antibodies, the design of hapten-carrier proteins considerably affects the specificity of the resultant antibody. The sodium periodate (NaIO_4_) oxidation method is the typical method for preparing the hapten-carrier protein conjugates for glycosides, which involves the oxidative cleavage of vicinal 1,2-diols of the sugar moieties to form imides with the amino group of lysine residues in the carrier proteins. Therefore, several anti-glycoside antibodies are prepared by the conjugates obtained from the NaIO_4_ oxidation method, which include paeoniflorin [34], solamargine [35], bacopaside I [36], saikosaponin a [37], liquiritin [38], and DZ [32, 33]. However, they tend to exhibit broad CR, especially with compounds containing similar aglycone parts. To obtain an MAb specific to DZ, Yusakul et al. recently designed hapten-carrier protein conjugates using the Mannich reaction, leading to the production of highly specific MAb to DZ (Table 3) [39]. Kitisripanya et al. investigated the effect of difference between the conjugates prepared via the NaIO_4_ oxidation method and the Mannich reaction on the specificity of the resultant PAb against miroestrol, which is a strong estrogenic compound produced in *Pueraria candollei* [40]. The PAb obtained from the hapten conjugate derived from the Mannich reaction exhibits higher specificity to miroesterol than that of the PAb obtained via the NaIO_4_ oxidation method, suggesting that the Mannich reaction is an important reaction for obtaining specific anti-hapten antibodies.

In addition to the method to prepare hapten-carrier protein conjugates, the number of hapten molecules bound to carrier proteins affects the specificity of antibodies. The hapten numbers are typically evaluated via matrix-assisted laser desorption/ionization time-of-flight mass spectrometry (MALDI-TOF–MS) using sinapinic acid as the matrix [41]. The relationship between the number of hapten molecules and antibody specificity has been investigated using a mercaptopropionic acid derivative of atrazine: high antibody titers with moderate antibody specificity are induced from 15–30 hapten molecules per carrier protein, while a lower number of hapten molecules exhibits a slower immune response with higher specificity [42]. This observation was also supported by the PAb against miroesterol reported by Kitisripanya et al. [40]. Recently, MAbs against the *Cephalotaxus* alkaloid harringtonine and pyrrolizidine alkaloid monocrotaline have been independently produced from their BSA conjugates using the NaIO_4_- and *N,N*′-carbonyldiimidazole-mediated methods, respectively, for their determination in plants. Both of the resultant MAbs exhibit extremely high specificity to both targets with high sensitivity, although only two hapten molecules are bound to BSA [43, 44].

### Utilization of antibody in icELISA depending on specificity

Antibodies exhibiting broad CR sometimes act as a useful and effective tool for recognizing a bioactive skeleton or a group of bioactive compounds because of the simultaneous determination by icELISA using the antibodies. Ginsenosides are the major compounds produced in ginseng and are classified into two groups according to their structure: 20(*S*)-protopanaxadiol and 20(*S*)-protopanaxatriol [45]. As they are considered as active compounds that exert various pharmacological activities of ginseng, such as tonic, immunomodulatory, antimutagenic, and anti-aging activities, they are focused as a target for quantitative/qualitative analysis in ELISA. With respect to 20(*S*)-protopanaxadiol, MAbs against G-Rb1 [46] and Rg3 [47] have been produced, while those against G-Re [48], G-Rg1 [49], and Rh1 and Rg2 [50] have been produced as representatives of 20(*S*)-protopanaxatriol for their specific determination. Interestingly, Morinaga et al. have produced MAb against G-Re exhibiting broad CR with G-Rd (76.2%) and G-Rg1 (70.9%) in addition to G-Re (100%) itself, enabling the development of icELISA for the simultaneous determination of the total ginsenosides in plant samples (Table 4) [51, 52].

**Table 4:** Chemical structures of representative ginsenosides and cross-reactivities (CRs) of MAb 4G10 and scFv used for simultaneous determination of total ginsenosides in plant samples by icELISA [51, 52, 62]

Ginsenosides	R_1_	R_2_	R_3_
Protopanaxatriol			
G-Re	H	Rha^1^–^2^Glc–O–	Glc–
G-Rg1	H	Glc–O–	Glc–
Protopanaxadiol			
G-Rb1	Glc^1^–^2^Glc–	H	Glc^1^–^6^Glc–
G-Rc	Glc^1^–^2^Glc–	H	Ara(f)^1^–^6^Glc–
G-Rd	Glc^1^–^2^Glc–	H	Glc–

Compound	CRs of MAb 4G10 [51]	CRs of GRe-scFv [62]
Protopanaxatriol		
G-Re	100	100
G-Rg1	70.9	67.2
Protopanaxadiol		
G-Rb1	< 0.009	< 0.009
G-Rc	< 0.009	< 0.009
G-Rd	76.2	73.5

### Sandwich ELISA

Sandwich ELISA has been widely accepted to exhibit higher specificity and wider working range as compared to the other types of ELISA. However, it is difficult to prepare two antibodies possessing different epitopes, especially for haptens because steric hindrance may disturb the antigen–antibody reaction because of the small size of the haptens. Therefore, true sandwich assays for hapten can be rarely developed, except for tacrolimus [53], angiotensin II [54], and naringin (Nar; which is a major flavonoid glycoside found in citrus fruits) [55]. Among these haptens, Nar is the smallest hapten with a molecular weight of 580 Da and the only plant secondary metabolite. Two hybridoma cell lines secreting different antibodies have been carefully screened, which enabled construction of a sandwich for Nar [55]. Interestingly, specificity to Nar in sandwich ELISA using two MAbs dramatically increases as compared with that in icELISA using a single MAb.

### ELISA using a recombinant antibody

**Fig. 6:**
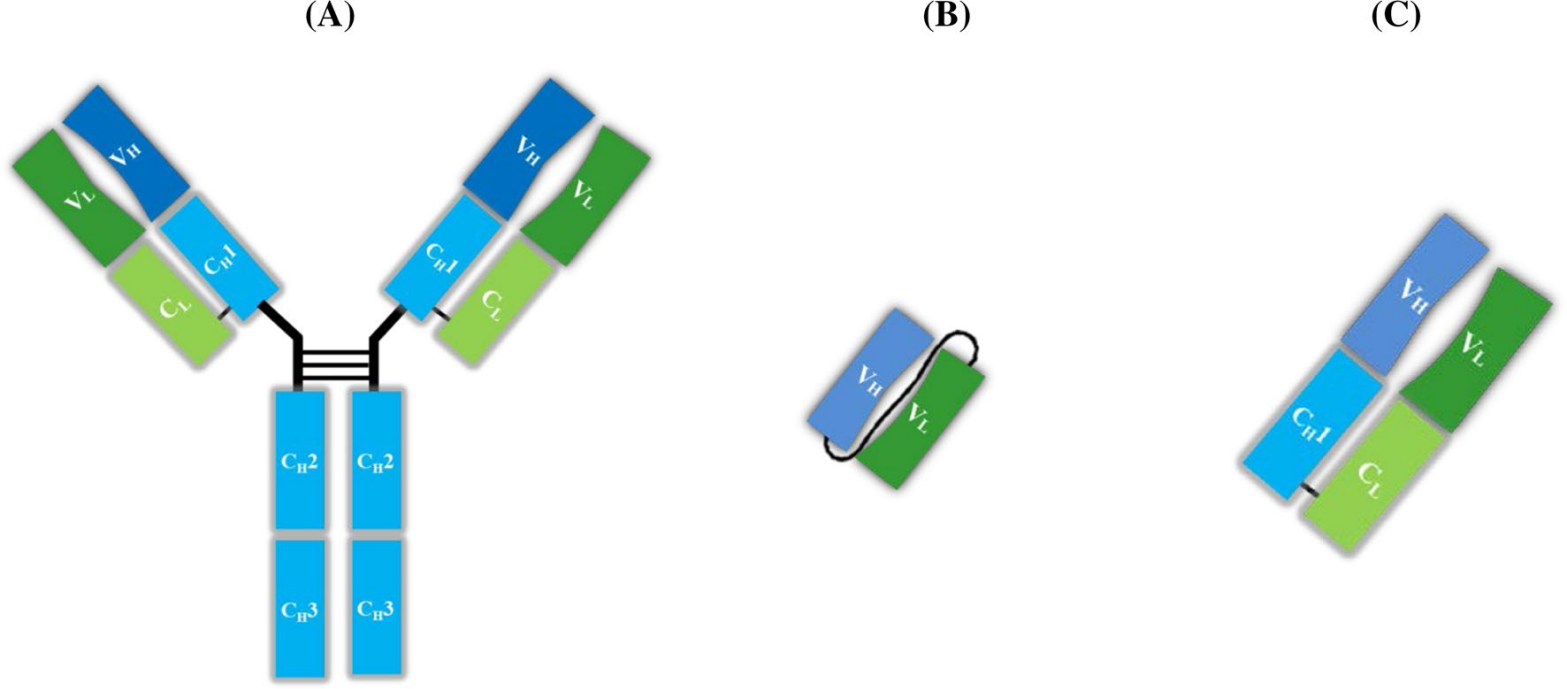
Schematic diagram of representative antibodies, IgG molecule (**a**), single chain variable fragment (scFv) antibody (**b**), and antigen-binding fragment (Fab) (**c**)

DNA recombinant technology has enabled the production of antibodies in *Escherichia coli* [56] and other organisms [57–60]; currently, recombinant antibodies (rAbs) have been reported to exhibit several advantages over conventional MAb and PAb in terms of production speed, the ability to modify properties through mutagenesis, and information on antibody–target interaction. Among rAbs, scFv and antigen-binding fragment (Fab) of an antibody are structurally independent units containing antigen-binding sites (Fig. 6). scFv consists of V_H_ and V_L_ chains with a flexible peptide linker of Gly and Ser, where the C-terminus of V_H_ is linked to the N-terminus of V_L_ and vice versa. Thus, their size decreases approximately to one-sixth of the original parental IgG molecule. Fab consists of a two-binding arm containing V_H_ and V_L_ chains, in addition to the constant regions of heavy (C_H_1) and light (C_L_) chains. They have become popular as a probe for ELISA because the original affinity and specificity of the original IgG molecule are maintained (Table 4).

A secondary antibody is required to detect rAbs in icELISA. The Fc region of immunoglobulin (MAb/PAb) is typically used as the epitope of secondary antibody for high versatility, while tags such as poly His-tag, T7-tag, and E-tag are commonly used as epitopes of secondary antibodies for rAb because they can be genetically incorporated into genes without disturbing the tertiary structure and activity of the rAb. Thus far, various scFvs against plant secondary metabolites have been constructed and expressed in *E. coli* to develop icELISA, including plumbagin [61], G-Re [62], DZ [63], wogonin glucuronide [64], and paclitaxel [65]. Similarly, Fab-based icELISA has been reported for artemisinin, which is produced from traditional Chinese herbal medicines, e.g., *Artemisia annua* L. and wogonin glucuronide, for their determination [66, 67]. They can be genetically engineered; therefore, fluorescent single-domain antibodies (fluobodies), chimera proteins of a green fluorescent protein (GFP), and an scFv also have been utilized in immunoassays. This combination always results in a 1:1 ratio between the fluorochrome and scFv, which overcomes the disadvantage of direct methods in immunoassays, i.e., deactivation of the antibodies with labeling enzymes. Furthermore, immunoassays using fluobodies enabled skipping of the time-consuming secondary antibody step with high sensitivity. Some studies have focused on these useful fluobodies to develop rapid and sensitive fluorescent-linked immunosorbent assays (FLISA) for plant secondary metabolites, including plumbagin [68] and G-Re [69]. In these reports, the fluobodies fusing scFv at the C-terminus of GFP were found to exhibit better affinity and sensitivity than those fusing at the N-terminus of GFP.

## Conclusion

To date, various methods for the quantitative or qualitative analysis of plant secondary metabolites have been developed because a lot of marketed drugs are generated from plant secondary metabolites, such as morphine (analgesic drug), vinblastine (antineoplastic drug), paclitaxel (antineoplastic drug), quinine (antimalarial drug), digitoxin (cardiotonic drug), and so on, and the accurate, sensitive, and selective evaluation of these drugs leads to safe clinical and general usages.

In this review, ELISA has been discussed in detail; it is representative of various analytical methods because of its several advantages over other analytical methods in terms of simplicity, cost efficiency, and selectivity. However, all types of ELISA exhibit more or less advantages and disadvantages. A barrier for further development of ELISA is the preparation of specific antibodies against the target hapten. Even in this advanced era, there are many important plant secondary metabolites for which antibodies are not available. ELISA would be more familiar to us if the antibody or antibody-mimicking probes that are alternatively used in ELISA could be obtained more easily.
