# Correction to: Enzyme-linked immunosorbent assay for the quantitative/qualitative analysis of plant secondary metabolites

**DOI:** 10.1007/s11418-017-1163-9

**Published:** 2018-01-05

**Authors:** Seiichi Sakamoto, Waraporn Putalun, Sornkanok Vimolmangkang, Waranyoo Phoolcharoen, Yukihiro Shoyama, Hiroyuki Tanaka, Satoshi Morimoto

**Affiliations:** 1 Department of Pharmacognosy, Graduate School of Pharmaceutical Sciences, Kyushu University, 3-1-1 Maidashi, Higashi-ku, Fukuoka, 812-8582 Japan; 2 Research Group for Pharmaceutical Activities of Natural Products using Pharmaceutical Biotechnology (PANPB), Faculty of Pharmaceutical Sciences, Khon Kaen University, Khon Kaen, 40002 Thailand; 3 Department of Pharmacognosy and Pharmaceutical Botany, Faculty of Pharmaceutical Sciences, Chulalongkorn University, 254 Phayathai Rd. Pathumwan, Bangkok, 10330 Thailand; 4 Department of Pharmacognosy, Faculty of Pharmaceutical Sciences, Nagasaki International University, 2825-7 Huis Ten Bosch, Sasebo, Nagasaki, 859-3298 Japan

## Correction to: Journal of Natural Medicines 10.1007/s11418-017-1144-z

The article Enzyme-linked immunosorbent assay for the quantitative/qualitative analysis of plant secondary metabolites, written by Seiichi Sakamoto, Waraporn Putalun, Sornkanok Vimolmangkang, Waranyoo Phoolcharoen, Yukihiro Shoyama, Hiroyuki Tanaka and Satoshi Morimoto, was originally published electronically on the publisher’s internet portal (currently SpringerLink) on 21 November 2017 without open access.

With the author(s)’ decision to opt for Open Choice the copyright of the article changed on [20 December 2017] to © The Author(s) 2017 and the article is forthwith distributed under the terms of the Creative Commons Attribution 4.0 International License (http://creativecommons.org/licenses/by/4.0/), which permits use, duplication, adaptation, distribution and reproduction in any medium or format, as long as you give appropriate credit to the original author(s) and the source, provide a link to the Creative Commons license and indicate if changes were made.

The original article was corrected.

